# From sepsis biomarker discovery to regulated *in vitro* diagnostics: evidence, certification status and implementation gaps

**DOI:** 10.3389/fmedt.2026.1916860

**Published:** 2026-09-22

**Authors:** Erika P. Plata-Menchaca, Enrique Hernández-Jiménez

**Affiliations:** 1 Medicine Department, Universitat Autònoma de Barcelona, Barcelona, Spain; 2 Medicine ICU Department, Hamad Medical Corporation, Doha, Qatar; 3 Loop Diagnostics S.L., StartUB!—University of Barcelona, Barcelona, Spain

**Keywords:** antimicrobial stewardship, biomarkers, endotoxin tolerance, host response, *in vitro* diagnostics, near-patient testing, sepsis—diagnostics

## Abstract

Sepsis remains a diagnostic challenge because early clinical manifestations are non-specific and microbiological confirmation often arrives too late to guide first-hour decisions. The 2026 Surviving Sepsis Campaign guidelines reinforce early clinical recognition using validated clinical scores, appropriate diagnostic strategies, and responsible antimicrobial use and de-escalation. Yet, initial management still frequently occurs under diagnostic uncertainty. Hundreds of biomarkers have been proposed to improve sepsis diagnosis, risk stratification and treatment guidance; however, only a limited subset has progressed from discovery to regulated *in vitro* diagnostic use. This Mini Review examines selected sepsis biomarkers and diagnostic platforms with emphasis on technologies that have reached certified or cleared IVD implementation, or that illustrate a credible route toward regulated use. We discuss conventional soluble biomarkers such as procalcitonin, C-reactive protein, interleukin-6 and lactate; emerging severity and host-response markers including presepsin, pancreatic stone protein, monocyte distribution width and mid-regional proadrenomedullin; transcriptomic and cellular host-response tests; and functional immune technologies such as the LPS-induced TNF-alpha release assay, also referred to as TARA. Rather than ranking biomarkers solely by diagnostic performance, we emphasize intended clinical use, biological rationale, sample type, turnaround time, regulatory status and workflow compatibility. The review highlights the persistent gap between biomarker discovery and clinical deployment, set against an evolving regulatory landscape shaped by the EU IVDR and the phased rollout of EUDAMED. Future sepsis diagnostics will likely move beyond isolated inflammatory analytes toward integrated host-response, near-patient and functional immune assays able to support early triage, risk stratification, antimicrobial stewardship and precision immune monitoring.

## Introduction

Sepsis is defined as life-threatening organ dysfunction caused by a dysregulated host response to infection (1). This definition places host biology, rather than pathogen detection alone, at the center of the syndrome. The global burden remains substantial, with 48.9 million sepsis cases and 11.0 million sepsis-related deaths estimated in 2017 (2). Yet the diagnostic problem remains operational as much as biological: emergency physicians and intensivists must treat or monitor high-risk patients before microbiological confirmation is available.

The 2026 Surviving Sepsis Campaign guidelines provide a contemporary clinical frame for biomarker evaluation. They focus on hospital, immediate prehospital and immediate post-hospital care, and incorporate antimicrobial stewardship through responsible antimicrobial use, diagnostic strategies and de-escalation (3). Therefore, a sepsis biomarker should not be judged only by analytical validity or area under the receiver operating characteristic curve. It should be judged by whether it can support a decision within the sepsis pathway.

General reviews consistently conclude that no single biomarker can replace clinical assessment, and that diagnostic value depends on timing, population, comparator and clinical context (4–7). However, those reviews largely rank candidate analytes by diagnostic accuracy and pay comparatively little attention to whether a biomarker exists as a regulated product with a defined intended-use population, validated thresholds and a workflow compatible with the sepsis pathway. Accordingly, this mini review focuses on the translational gap between biomarker discovery and regulated IVD deployment, organizing representative technologies by regulatory status, intended use and workflow feasibility rather than by diagnostic performance alone. It highlights representative biomarkers and platforms that illustrate different routes to implementation, including soluble protein assays, hematological parameters, transcriptomic host-response tests, cellular biophysical technologies and functional immune assays. The selected technologies are summarized in Table 1 and integrated within the translational framework shown in Figure 1.

**Table 1:** Regulated or commercially available diagnostic technologies for sepsis biomarker assessment.

Biomarker/test	Biological domain	Technology/platform	Intended clinical use	Regulatory status	Main implementation limitation
Procalcitonin, PCT	Systemic inflammatory response associated with bacterial infection	Quantitative automated immunoassays in serum or plasma; multiple manufacturers and laboratory platforms	Adjunctive assessment of bacterial infection and sepsis risk; support for antibiotic initiation or discontinuation in specific populations and according to assay-specific claims	Multiple CE marked IVD assays in Europe; several FDA cleared assays with platform-specific indications in the United States	Concentrations depend on sampling time and clinical context and may be affected by surgery, trauma, renal dysfunction and non-infectious inflammation; not a stand-alone sepsis test
CRP, IL-6 and lactate	Acute phase inflammation, cytokine activation and tissue hypoperfusion or metabolic stress	Automated clinical chemistry, immunoassay or blood gas platforms using serum, plasma or whole blood	Assessment of inflammation, disease severity, tissue perfusion and adjunctive clinical risk stratification	Broad IVD availability; sepsis-specific claims vary between analytes, assays and platforms	Limited specificity for sepsis and limited ability to distinguish infectious from non-infectious inflammation or classify immune dysfunction
Presepsin	Soluble CD14 subtype, sCD14-ST, associated with innate immune and phagocytic activation	PATHFAST™ chemiluminescent enzyme immunoassay using whole blood or plasma, PHC Corporation, Tokyo, Japan	Aid in sepsis diagnosis and prognosis, assessment of severity and risk stratification of critically ill septic patients	Available in Europe as an IVD for near patient testing; no FDA clearance for a presepsin sepsis indication was identified	Requires access to the dedicated PATHFAST platform; reference intervals and decision thresholds may vary between populations, settings and laboratories
MR-proADM	Endothelial dysfunction, vascular integrity and systemic organ stress	Automated immunofluorescence assay, such as B·R·A·H·M·S MR-proADM KRYPTOR, Thermo Fisher Scientific	Prognostic and severity assessment and support for level-of-care decisions in patients with suspected infection or sepsis	CE marked assay availability in Europe; no FDA 510(k) clearance was identified	Primarily a severity and prognostic biomarker rather than a test for establishing the infectious etiology or confirming sepsis
Pancreatic stone protein, PSP	Host stress and inflammatory response associated with infection and sepsis development	Rapid semi-quantitative nanofluidic immunofluorescence assay using EDTA whole blood, IVD CAPSULE PSP on abioSCOPE®, Abionic SA, Epalinges, Switzerland	Aid in the early detection of sepsis manifesting within 3 days after testing in adult patients at high risk of sepsis presenting to the ICU	FDA 510(k) K240041; IVD CAPSULE PSP certified under the EU IVDR according to the manufacturer	FDA intended use is restricted to high-risk adult ICU patients; requires a dedicated analytical platform and should be interpreted with clinical and laboratory findings
Monocyte distribution width, MDW	Morphological variability of monocytes associated with cellular activation during infection	Automated hematology parameter reported with the complete blood count and differential using the Early Sepsis Indicator application on compatible Beckman Coulter DxH analyzers	Aid in identifying adult emergency department patients with sepsis or at increased risk of developing sepsis	FDA 510(k) K181599 for the UniCel DxH 800 with Early Sepsis Indicator; CE marked availability on the DxH 900 is reported by the manufacturer	Restricted to compatible hematology analyzers; thresholds and diagnostic pathways require local validation, and the result remains adjunctive rather than diagnostic on its own
SeptiCyte® RAPID	Two-gene host response mRNA signature based on PLAC8 and PLA2G7 expression	Automated RT-qPCR using whole blood on the Biocartis Idylla™ System, Immunexpress, Inc., Seattle, WA, USA; platform manufactured by Biocartis NV, Mechelen, Belgium	Aid in differentiating infection-positive systemic inflammation, or sepsis, from infection-negative systemic inflammation in patients suspected of sepsis on the first day of ICU admission	FDA 510(k) K203748, with additional EDTA-compatible cartridge clearance under K232095; CE marked IVD availability in Europe	FDA intended use is population and setting specific; requires dedicated molecular instrumentation and compatible blood collection and cartridge workflows
TriVerity™	Twenty-nine host response mRNA transcripts and three housekeeping transcripts	Automated quantitative RT-LAMP using PAXgene whole blood collection tubes and single-use cartridges on the Myrna™ Instrument, Inflammatix, Inc., Sunnyvale, CA, USA	Aid in differentiating bacterial infection, viral infection and non-infectious illness and estimating the likelihood of mechanical ventilation, vasopressor use or renal replacement therapy within 7 days in adult emergency department patients	FDA 510(k) K241676; European regulatory status was not identified	Restricted to adult emergency department patients; requires PAXgene collection and a dedicated proprietary molecular platform; implementation and clinical outcome evidence remain comparatively recent
IntelliSep®	Biophysical and mechanical properties of activated leukocytes	Semi-quantitative deformability cytometry using EDTA whole blood on the Cytovale System, Cytovale, Inc., South San Francisco, CA, USA	Aid in the early detection of sepsis with organ dysfunction manifesting within 3 days after testing in adult patients with signs or symptoms of infection presenting to the emergency department	FDA 510(k) K220991; European CE status was not established in the sources reviewed	Requires a dedicated cellular analysis platform; restricted to adult emergency department use and does not identify the causative pathogen
SeptiLoop®, commercial implementation of the TNF-α release assay, TARA	Functional innate immune responsiveness measured through inducible TNF-α release after *ex vivo* LPS stimulation; reflects endotoxin tolerance and immune hyporesponsiveness	Manual near patient stimulation of heparinized whole blood with LPS followed by qualitative TNF-α lateral flow detection, Loop Diagnostics S.L., Barcelona, Spain	Aid in identifying patients at risk of induced immunosuppression during suspected bacterial bloodstream infection, including sepsis	CE marked under the EU IVDR as a Class C near patient test; UKCA certified; FDA clearance was not identified	Requires controlled *ex vivo* stimulation, incubation temperature, timing and readout conditions.
Sepsis ImmunoScore™	Multimodal integration of clinical variables and sepsis-associated laboratory biomarkers	Locked AI/ML probability-random-forest Software as a Medical Device using up to 22 predetermined EHR inputs, including demographics, vital signs, hematology and chemistry values and sepsis biomarker concentrations; Prenosis, Inc., Chicago, IL, USA	Adjunctive assessment of the presence of or progression to sepsis within 24 h in adults admitted to the emergency department or hospital with suspected sepsis and a blood culture ordered; outputs a risk score and Low, Medium, High or Very High category	FDA *de novo* DEN230036, Class II, product code SAK (8); regulated as SaMD rather than as an IVD assay	One-time assessment requiring a blood-culture order; not validated in patients younger than 18 years or in specific settings such as ICU or labor and delivery; dependent on EHR data completeness and should not be used as a stand-alone diagnostic

CBC, complete blood count; CRP, C-reactive protein; ED, emergency department; FDA, U.S. Food and Drug Administration; IL-6, interleukin-6; IVDR, *in vitro* Diagnostic Medical Devices Regulation; LPS, lipopolysaccharide; MDW, monocyte distribution width; MR-proADM, mid-regional proadrenomedullin; PCT, procalcitonin; PSP, pancreatic stone protein; TARA, TNF-alpha release assay.

**Figure 1:**
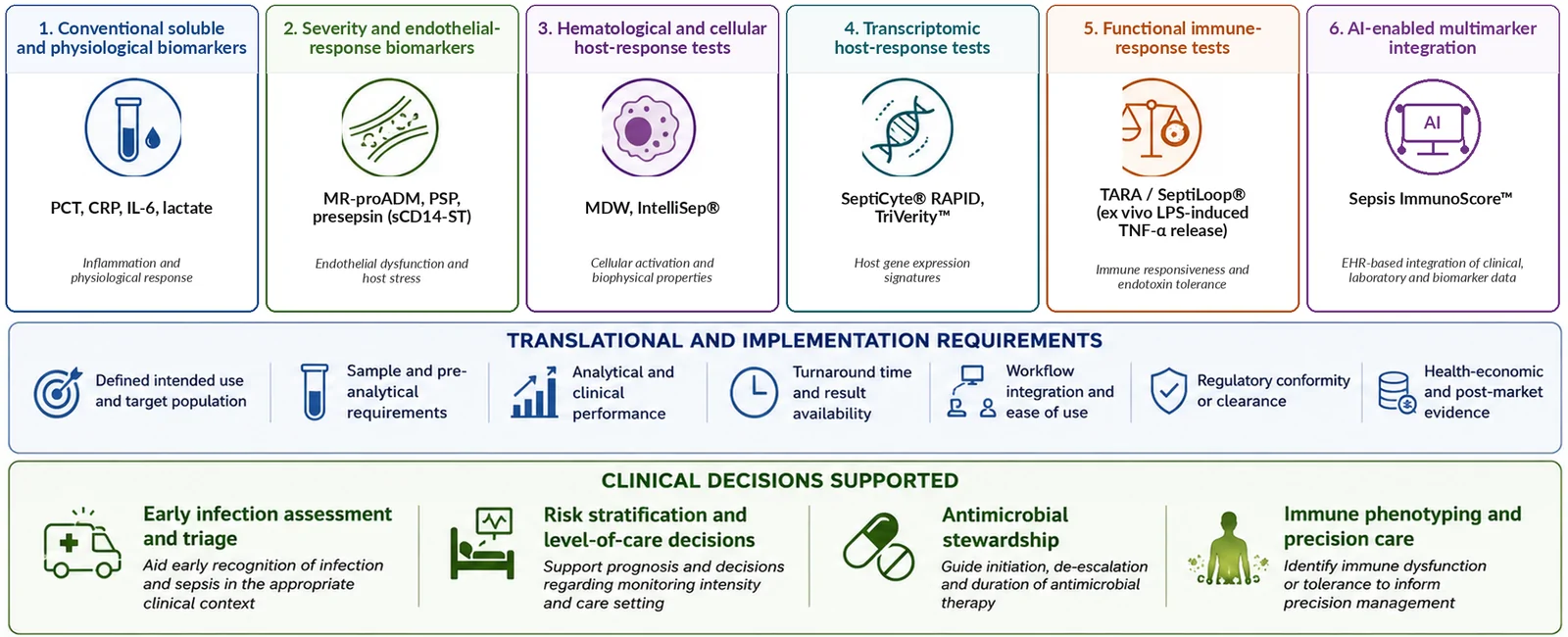
Translational framework for the clinical implementation of sepsis biomarker-based diagnostic technologies**.** Representative technologies are organized according to the biological signal measured, ranging from soluble and physiological biomarkers to hematological, cellular, transcriptomic, functional immune-response assays and AI-enabled multimarker integration. These categories are complementary rather than sequential and may support different clinical decisions, including early infection assessment, risk stratification, antimicrobial stewardship and immune phenotyping. Translation into clinical practice requires a clearly defined intended use and target population, validated analytical and clinical performance, acceptable turnaround time, workflow compatibility, appropriate regulatory conformity or clearance, and evidence of clinical and health-economic value.

### Review methodology

The review question was developed using a PICO-informed framework focused on patients with suspected infection or sepsis, diagnostic and risk-stratification technologies based on physiological or host-response biomarkers, available comparators, and clinically relevant outcomes. A targeted search was performed in PubMed/MEDLINE, Google Scholar, ScienceDirect, SpringerLink and Wiley Online Library, supplemented by searches of ClinicalTrials.gov, the WHO International Clinical Trials Registry Platform, official regulatory sources including the FDA and European Commission/EUDAMED, manufacturer instructions for use and publicly available technical documentation. Search concepts combined ’sepsis’ or ’suspected infection’ with ‘biomarker,’ ‘diagnostic,’ ‘*in vitro* diagnostic,’ ‘host response,’ ‘point-of-care,’ ‘near-patient,’ individual biomarker or platform names, ‘artificial intelligence,’ ‘machine learning,’ ‘FDA,’ ‘CE-IVD’ and ‘IVDR.’ The search covered January 2010 through May 2026, while earlier landmark studies were retained when required to establish biological rationale.

Eligible sources included peer-reviewed human studies, systematic reviews and meta-analyses, guidelines, official regulatory decisions and publicly available instructions for use that described sepsis-related diagnostic technologies and reported information concerning biological rationale, clinical performance, intended use, specimen type, turnaround time, workflow, regulatory status or implementation limitations. Priority was given to technologies with regulated or commercial IVD implementation or a credible documented pathway toward clinical deployment. Animal-only or basic discovery studies without human validation, technologies without a defined clinical use or translational pathway, case reports, conference abstracts with insufficient information, duplicate publications and unpublished or proprietary company data were excluded. The final included evidence and extracted characteristics were cross-checked by both authors, with differences resolved by consensus. The same extraction domains were applied to every technology: intended use, biological signal, specimen, analytical platform, turnaround time, regulatory status, workflow requirements and principal implementation limitations.

### Conventional soluble biomarkers: mature IVDs with limited specificity

Procalcitonin (PCT), C-reactive protein (CRP), lactate and interleukin-6 (IL-6) remain common reference points for sepsis biomarker discussions. Their strengths are availability, analytical standardization and compatibility with laboratory workflows. PCT has multiple regulated assay formats and has been used to support bacterial infection assessment, risk stratification and antibiotic discontinuation in selected contexts (9, 10). Lactate is not sepsis-specific, but it remains central to severity assessment and shock recognition.

The main limitation of these markers is biological non-specificity. CRP and IL-6 reflect inflammation rather than infection-specific immune failure. PCT is more closely associated with bacterial infection, but its interpretation is affected by disease context, timing, renal dysfunction, surgery, trauma and immunological heterogeneity. These biomarkers are useful components of clinical assessment, but they do not fully capture the host-response complexity embedded in the Sepsis-3 definition.

### Emerging soluble and hematological IVD biomarkers

A second group of biomarkers aims to improve diagnosis, prognosis or early triage by measuring more specific host-response or cellular signals. Presepsin, a soluble CD14 subtype, and mid-regional proadrenomedullin (MR-proADM), a marker linked to endothelial dysfunction and vascular stress, have shown good diagnostic performance in a meta-analysis of adult patients with sepsis, with MR-proADM slightly superior in that analysis (11). In practice, however, presepsin is generally framed as an infection or sepsis support marker, whereas MR-proADM is often more compelling for severity, deterioration risk and level-of-care decisions.

Pancreatic stone protein (PSP) has moved into regulated device development and has been associated with diagnosis or prognosis in high-risk infection settings (12–14). The FDA cleared the IVD CAPSULE PSP on the abioSCOPE® system (Abionic SA, Epalinges, Switzerland) as an aid in early detection of sepsis in adult ICU patients at high risk of sepsis (15). Monocyte distribution width (MDW) illustrates another implementation model. It reflects monocyte morphological variability and can be generated within hematology analyzer workflows. A systematic review and meta-analysis support its use as an early sepsis screening marker, especially in emergency settings (16), and the FDA cleared the Early Sepsis Indicator application on the UniCel DxH 800 Cellular Analysis System (Beckman Coulter, Inc., Brea, CA, USA) for adult emergency department patients (17). The main advantage is workflow integration, but value still depends on prevalence, threshold, intended use and the action taken after the result.

### Molecular and cellular host-response diagnostics

Host-response molecular diagnostics represent a conceptual shift from pathogen detection or isolated inflammatory proteins to classification of the patient's biological response. SeptiCyte® RAPID (Immunexpress, Inc., Seattle, WA, USA), run on the Idylla™ platform (Biocartis NV, Mechelen, Belgium), is an FDA-cleared RT-qPCR host-response assay intended to help differentiate infection-positive sepsis from infection-negative systemic inflammation in ICU patients (18). Laboratory evaluations and clinical studies support the concept that host gene expression can provide information partly independent of conventional markers (19, 20).

TriVerity™ (Inflammatix, Inc., Sunnyvale, CA, USA) extends this logic through a 29-mRNA host-response signature designed to differentiate bacterial infection, viral infection and non-infectious illness and to estimate risk of severe illness within seven days (21). Recent analytical evaluation supports the feasibility of rapid host mRNA measurement on an automated RT-LAMP platform (22). IntelliSep® (Cytovale, Inc., South San Francisco, CA, USA) uses leukocyte biophysical properties, measured by deformability cytometry, to classify the cellular host response in adult emergency department patients with suspected infection (23). Clinical evidence supports its potential for ED risk stratification (24). These technologies are important because they operationalize a central idea in modern sepsis biology: the host response itself can be a diagnostic analyte.

### Functional immune assays and TARA

Functional immune assays add another layer by measuring what immune cells can do after stimulation, rather than only measuring basal analyte concentrations. Endotoxin tolerance is a well-established model of leukocyte reprogramming in sepsis, defined by reduced responsiveness to a lipopolysaccharide challenge after prior exposure to endotoxin or inflammatory stress (25). Immune monitoring studies support the clinical relevance of impaired monocyte antigen presentation, reduced HLA-DR expression and attenuated *ex vivo* TNF-alpha responses (26–30).

TARA refers to an LPS-induced TNF-alpha release assay performed on whole blood to assess inducible innate immune responsiveness. In a 2024 study, patients with positive blood cultures showed significantly lower TNF-alpha production after LPS challenge than patients with negative blood cultures, supporting the link between bacteremia-associated sepsis and functional innate immune suppression (31). SeptiLoop® (Loop Diagnostics S.L., Barcelona, Spain) is a commercial implementation of TARA. It is a qualitative near-patient IVD in which heparinized whole blood is stimulated with LPS, followed by lateral flow detection of TNF-alpha to identify endotoxin tolerance status (32).

### AI-enabled multimarker integration

Artificial intelligence and machine-learning approaches offer a complementary route to sepsis diagnosis by integrating multiple weak or partially independent signals rather than relying on a single biomarker. Model inputs may include demographic characteristics, vital signs, routine hematological and biochemical measurements and sepsis-specific biomarkers, with the combined information converted into risk scores or clinically interpretable categories (33). A regulated example is the Sepsis ImmunoScore (Prenosis, Inc., Chicago, IL, USA), an FDA-authorized AI/ML software device that uses up to 22 electronic-health-record inputs, including procalcitonin, C-reactive protein and lactate, to support assessment of the presence of or progression to sepsis within 24 h in adults undergoing evaluation for suspected sepsis (8). These systems may improve discrimination and provide clinical context for individual biomarker results, and future models could potentially combine soluble, transcriptomic, cellular and functional immune measurements. However, AI should not be regarded as a replacement for validated biological measurement. Its clinical value depends on representative development data, transportability between institutions, management of missing information, calibration, interoperability, explainability, avoidance of alert fatigue and prospective demonstration that model-guided actions improve patient outcomes (34). Moreover, these products are generally regulated as Software as a Medical Device rather than as conventional IVD assays, creating a related but distinct translational pathway. The most plausible future model may therefore involve regulated biomarkers providing reproducible biological signals and AI integrating those signals with the wider clinical context.

### Regulatory and implementation gaps

The distinction between biomarker evidence and regulated diagnostic use is essential. A molecule may be biologically plausible and statistically associated with sepsis, yet still lack a regulated product, an intended-use population, validated thresholds, instructions for use, workflow compatibility or evidence that acting on the result improves outcomes. In Europe, IVDR has increased the evidentiary and quality requirements for IVDs, especially for higher-risk assays and near-patient testing (35). EUDAMED has further increased regulatory transparency since its first four modules became mandatory on 28 May 2026 (36).

Regulatory status should therefore be reported by jurisdiction and by intended use. CE-IVD or IVDR certification, FDA clearance, TGA inclusion or Health Canada licensing do not necessarily imply identical claims, populations or workflows. For sepsis biomarkers, this matters because the same signal may support different decisions: early recognition, bacteremia risk, prognosis, de-escalation, ICU triage, immune phenotyping or monitoring.

### Discussion and future perspectives

The biomarker field is moving from isolated inflammatory analytes toward multi-layered host-response diagnostics. Conventional markers remain useful because they are accessible and familiar, but their limitations explain why newer technologies are emerging. Soluble severity markers, hematology-derived cellular parameters, transcriptomic classifiers, leukocyte biophysical assays and TARA are not interchangeable. They answer different clinical questions and rest on distinct technology platforms, such as automated immunoassays, hematology analyzers, RT-qPCR and RT-LAMP molecular systems, deformability cytometry and near-patient lateral-flow readouts. Each platform has different demands for instrumentation, automation and operator expertise that directly shape feasibility at the point of care. The key question is not whether a biomarker is associated with sepsis, but whether a regulated test improves clinical decision-making that clinicians actually face: whether to admit, escalate, monitor more closely, start or discontinue antimicrobials, or identify a patient with immune dysfunction who may require a different management strategy. Future studies should evaluate biomarkers in decision-specific pathways, report intended-use populations clearly and include workflow feasibility, turnaround time, health-economic impact and post-market evidence. In this framework, transcriptomic host-response tests, leukocyte biophysical assays and TARA may complement rather than replace conventional biomarkers. Their clinical value will depend on whether they reduce diagnostic uncertainty where that uncertainty affects treatment decisions, resource use and patient risk.

## Limitations

This review has several limitations. Formal risk-of-bias assessment, evidence grading, and quantitative meta-analysis were beyond its scope. Comparisons between diagnostic technologies should be interpreted cautiously because of heterogeneity in study populations, sepsis definitions, clinical settings, reference standards, and endpoints. Regulatory status is jurisdiction- and product-specific and may change over time. In addition, while evidence is rapidly expanding, many emerging diagnostic and AI-enabled technologies still require prospective studies to confirm their impact on clinical decision-making and patient outcomes.
